# Addressing Substance Use Utilizing a Community-Based Program among Urban Native American Youth Living in Florida

**DOI:** 10.3390/genealogy4030079

**Published:** 2020-07-23

**Authors:** Rose Wimbish-Cirilo, John Lowe, Eugenia Millender, E. Roberto Orellana

**Affiliations:** 1 National Institutes of Health (NIH)/National Institute on Drug Abuse (NIDA), Florida Atlantic University, Boca Raton, FL 33431, USA; 2 Nursing Department, Broward College, Miramar, FL 33024, USA; 3 College of Nursing, Florida State University, Tallahassee, FL 32306, USA; 4 School of Social Work, Portland State University, Portland, OR 97201, USA

**Keywords:** substance use prevention, Native-Reliance, urban Native American youth, community-based program

## Abstract

This study was conducted in Florida among two urban Native American youth programs that are sponsored by urban Native American community organizations. Convenience and snowballing were used as a sample recruitment strategy. Assignment to the experimental condition (UTC) and the control condition (SE) was established by randomizing the two community youth program sites to the two conditions. Utilization of a culturally relevant theory, Native-Reliance, guided the intervention approach for the prevention of substance use among urban Native American youth. Results of this study provided evidence that a culturally based intervention was significantly more effective for the reduction of substance use interest and general well-being than a non-culturally based intervention for urban Native American youth. Prevention programs for urban Native American early adolescent youth that utilize Native American strengths, values, and beliefs to promote healthy behavior and reduce the harm associated with high-risk behaviors such as substance use are strongly recommended.

## 1. Introduction

The prevalence of alcohol and drug use among urban Native Americans in the United States (U.S.) is detrimental to Native American health and well-being. Evidence from research identifies that Native American people who live in urban areas away from traditional tribal settings experience high rates of alcohol and drug use (SAMHSA and CBHSQ 2015). Additionally, there is a lack of understanding in relation to urban Native American worldview and cultural values among health care professionals (Gone and Trimble 2012; Goodkind et al. 2011; Knibb-Lamouche and IOM 2012). This has impeded the capacity of health care professionals to develop culturally relevant alcohol and drug use health prevention interventions for urban Native American youth.

Within the United States, there are a considerable number of urban Native American people living within Florida who are vulnerable to engaging in alcohol and drug use. According to the U.S. Census Bureau (2016), Florida ranks as one of the leading states consisting of more than 100,000 Native American citizens. Incidence rates for accidental death, domestic violence, suicide, incarceration, illness, and disease associated with alcohol and drug use among urban Native Americans in the United States are rising steadily (USDHHS 2014; UIHI 2014; SAMHSA and CBHSQ 2015). In Florida, urban Native American vulnerability to alcohol and drug use during adolescence can negatively impact the healthy existence of this population across the life span. National demographic data from the last six years indicate that urban Native American are one of the highest-ranking groups for the incidence of alcohol and drug use per 100,000 citizens (SAMHSA 2013; SAMHSA and CBHSQ 2015).

Morbidity and mortality rates among urban Native Americans who engage in alcohol and drug use are rising steadily (Goodkind et al. 2011; Hodge and Nandy 2011; Urban Indian Health Commission 2015). Incidence rates of alcohol and drug use disorders are higher in people age 12 and older (9.3%), which ethnically categorizes the population with one of the highest rates of any other ethnic group living in the US (SAMHSA 2015). Native American mortality rates per 100,000 Native American persons versus the general US population are higher for alcohol-related liver disease/cirrhosis (5.5% versus 1.5%) and alcohol/drug-induced death (15.5% versus 19.8%) (CDC 2014a, 2014b, 2017; CDC and Heron 2019; CDC et al. 2019a). Conversely, Native American alcohol/drug-induced death rates may vary from state to state and increase by 1.5 to 3-fold higher than the general population (CDC 2017; CDC et al. 2019a, 2019b; CDC and Heron 2019; Northwest Portland Area Indian Health Board 2019). These increasing rates indicate there is a need to address and study alcohol and drug use prevention among urban Native American youth within Florida.

Various studies indicate alcohol and drug use among Native Americans are linked to comorbidities such as hepatitis, chronic liver disease, cirrhosis, renal failure, gastrointestinal, cancer, cardiomyopathy, and brain impairment disorders (Allen et al. 2014; CDC 2015; SAMHSA and CBHSQ 2015). Alcohol and drug use among urban Native Americans is a leading health disparity contributing to the increasing incidence of morbidity and mortality in this population (Office of Disease Prevention and Health Promotion 2020; CDC 2013). Therefore, the acknowledgement of cultural values, beliefs, and practices is needed in order to implement culturally relevant and appropriate services to impact the potential reduction of health disparities among urban Native American populations.

Many Native American people perceive health as balance, harmony, and connectedness (Hodge and Nandy 2011; Lowe 2002). Cultural ways of knowing and being among Native Americans encompass a holistic worldview that supports health and well-being (Hodge and Nandy 2011; Lowe and Struthers 2001; Lucero 2009). There is often a minimal understanding of how health and well-being among Native Americans are interrelated to their unique cultures, extended families, and tribal societies (Simmons 2014; UIHI 2014; Wiechelt and Okundaye 2012). This indicates there is a need to address health needs such as Native American alcohol and drug use prevention from a cultural perspective to decrease the likelihood of health disparities among Native American populations.

Currently, there are very few evidenced-based interventions for the prevention of alcohol and drug use that involve culturally tailored approaches for Native American adolescent youth (Croff et al. 2014; Dickerson et al. 2012; Donovan et al. 2015; Kulis et al. 2011; Lowe et al. 2012; Patchell 2011). Even fewer evidence-based interventions have been implemented for alcohol and drug use among urban Native American adolescent youth (Kulis et al. 2011; Lowe et al. 2016). This finding identifies there are limited culturally relevant evidence-based interventions among urban Native American people to reduce and prevent the incidence of alcohol and drug use. Health professionals need to understand the health perspectives of urban Native Americans in order to provide alcohol and drug use prevention community-based programs. Addressing alcohol and drug use prevention among urban Native Americans involves understanding Native American non-Western health perspectives. Implementing interventions that prevent alcohol and drug use among urban Native American youth also requires the importance of cultural safety and humility at both the individual interventionist and institutional levels where development and testing of innovative approaches occur (Hook et al. 2013; Papps and Ramsden 1996). The research team who are developing research designs and the individuals responsible for delivering the intervention must acknowledge and address their own biases, attitudes, assumptions, stereotypes, prejudices, structures, and characteristics that may affect the quality of the intervention being implemented. This includes a process of ongoing self-reflection and self-awareness where there is accountability for providing culturally safe care that should be defined by the urban Native American community and individuals being impacted by the intervention (Darroch et al. 2017).

### Theoretical Framework

**Figure 1.**
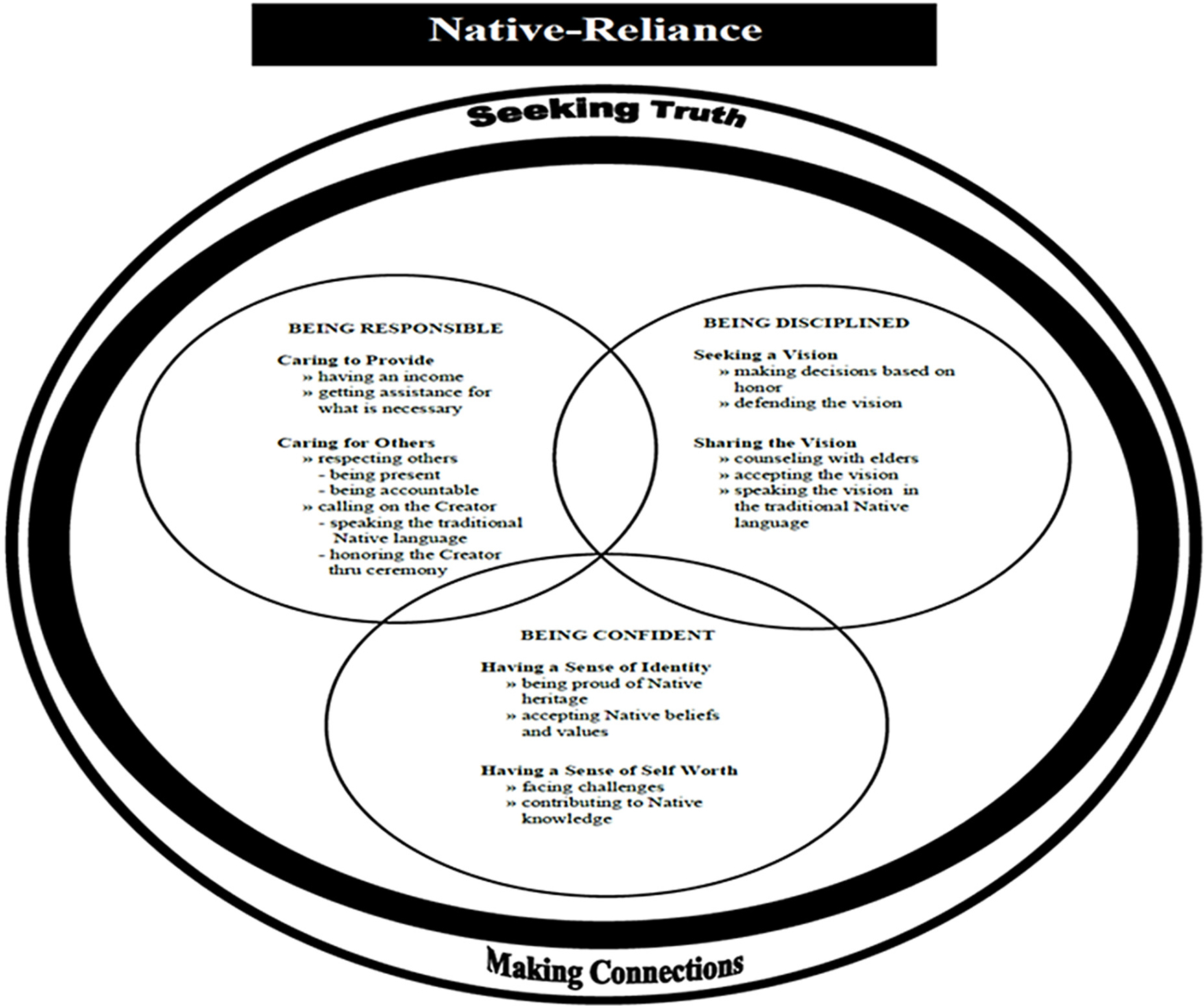
Native-Reliance Theory and guiding framework.

The Native-Reliance theoretical framework and model guided this study by providing a cultural perspective and foundation from which to examine alcohol and drug use prevention among urban Native American youth in a meaningful way. Native-Reliance is conceptualized as a continuous, yet all-encompassing process representing the holistic worldview of Native American ways of knowing and being, cultural identity, health perspectives, and well-being (Lowe 2017). Three internally linked circles and cultural themes (core qualities) of being responsible, being confident, and being disciplined embody the interrelationship between the overarching cultural domains of seeking truth and making connections. The Native-Reliance theoretical framework and model has guided several intervention studies (Lowe et al. 2016, 2019; Patchell et al. 2015). The Native-Reliance theoretical framework posits that when Native American people remain faithful to their own cultural practices, they exhibit behaviors consistent with healthy living through resources that support wellbeing (Lowe 2017; Lowe et al. 2012, 2019). Figure 1 depicts the model as an interrelationship of the three core qualities and two cultural themes of Native-Reliance Theory and guiding framework.

## 2. Method

### Research Design

This study consisted of a one-year plan using a two-condition design in which the culturally-based intervention, Urban Talking Circle (UTC), was compared to standard care/standard education, Be A Winner/Drug Abuse Resistance Education (SE). Approval was obtained from an urban Native American community-based organization along with Institutional Review Board approval from the university initiating the research. Participants who were recruited included 100 urban Native American 10–12-year-old youth living in Florida. Assent and parental/guardian consents were obtained for each participant. The two participating urban Native American community-based youth programs were randomized to the two conditions and were situated approximately 150 miles from each other. Therefore, the potential for cross contamination was limited between the two selected programs.

## 3. Urban Talking Circle (UTC) Intervention

The UTC is a 10-session manual-based intervention designed for urban Native American youth ages 10–12 years old. The overall goal of the intervention was to reduce interest in substance use. The youth participants engaged in a group led by a counselor and urban Native American cultural expert that met for a 45-min session in the format of a talking circle once a week over a 10-week period. Talking circles among Native Americans is a coming together and a place where stories are shared in a respectful manner in a context of complete acceptance by participants and used to celebrate the sacred interrelationship that is shared with one another and with their world (Lowe et al. 2012; Simpson 2000). The talking circle process is a unique instructional approach that can be used to stimulate cultural awareness while fostering respect for individual differences and facilitating group cohesion. The symbol of the circle holds a place of special importance in Native American beliefs and the significance of the circle has always been used as a way of bringing people of all ages together for the purposes of teaching, listening, and learning (Wolf and Rickard 2003). Native Americans have always believed that healing and transformation should take place in the presence of the group (Lowe et al. 2016). Through the use of the talking circle, Native American youth gain support and insight from each other along with a sense of belonging. Talking circles are a traditional Native American format for educating and providing a way to pass on knowledge, values, and culture (Lowe and Wimbish-Cirilo 2016). Each participant committed to the group to be respectful by maintaining confidentiality of what was shared. The manual integrated cultural concepts related to Native-Reliance values and both English and local urban tribal languages were included.

## 4. Standard Substance Abuse Education (SE)

The SE condition participants were enrolled in a program entitled “Be A Winner”, which was chosen as the standard condition because it is the usual program provided to students within the school systems where the participants attend school. The participants indicated they had not yet received the program in their schools. The program is a revision of the “Drug Abuse Resistance Education” (D.A.R.E.) program designed as a youth substance abuse education program that promotes a school/law partnership approach to substances/drug education (DARE 2016). It provided the police officer implementing the program with an organized curriculum and workbook to present substance/drug education within a classroom setting that occurred for 45-min sessions. Each session occurred once a week for 10 weeks.

### 4.1. Sample and Setting

This study was conducted in Florida among two urban Native American youth programs sponsored by a Native American community-based organization. Convenience and snowballing were used as a sample recruitment strategy. Food and snacks were provided at each session which assisted in the recruitment and retention of participants. An equal opportunity of participant assignment to the experimental condition (UTC) and the control condition (SE) was established by randomizing the two community-based youth program sites to the two conditions. A sample size was calculated by use of G* Power 3 computer software (Version 3.1.9.2) and the total sample size included 100 male and female urban Native American early adolescent youth between the ages of 10–12 years. Each study condition included 50 participants.

### 4.2. Measurements

Pre-intervention (baseline) and post-intervention were the two data collection points. A demographic instrument was included which asked for several routine socio-demographic variables. In addition, the participants were asked if they had been involved in any prevention programs. The Native-Reliance Questionnaire, Indigenous—Global Assessment of Individual Needs (I-GAIN), and the Native American Alcohol Measure for Youth (NAAMY) were administered at the same two data collection points.

The Native-Reliance Questionnaire, which includes 24 items rated using a Likert scale, was used to evaluate the presence of Native-Reliance and has a test–retest reliability coefficient alpha of 0.84. The I-GAIN includes an overall Drug Use Interest Scale and previous studies with youth have found good reliability in test–retest situations (Lowe et al. 2019). Measures that assess for alcohol use interest among Native American youth are very limited and scarce. The NAAMY was recently developed by six expert Native American and Indigenous researchers which includes an over Alcohol Use Interest Scale. Face and content validity were recently assessed and confirmed by six expert researchers along with a test-retest reliability coefficient alpha of 0.86.

## 5. Results

A total of 100 urban Native American ages 10–12 years were recruited for this study where 50 youth participated in the UTC group and 50 youth participated in the SE group. Groups were randomly assigned to the UTC condition and the SE condition. Participants completed all of the sessions and all pre and post measurements. A MANOVA was conducted to determine if there were significant differences between the scores on all measures at pre-intervention (baseline) and post-intervention of the two condition groups (UTC and SE).

The participants identified themselves as southeastern urban Native American youth living in Florida with heritage to the various tribes located in the southeastern region of the US. Both males (n = 49) and females (n = 53) were included as participants and they ranged in age from 10 to 12, with a mean age of 10.8 years (SD = 4.52). Participants reported 98.1% attendance and 1% non-attendance at all of the study intervention sessions. The sample’s mean level of education by grade was 5.8. The majority of participants lived with the following relatives: father/mother, 47.6%, mother, 15.5%, grandfather/grandmother, 8.7%, father/grandmother, 5.8%, and mother/grandmother, 5.9%, respectively. Mean scores for participants with female siblings were 1.01% and with male siblings were 1.03.

### 5.1. Native-Reliance

**Figure 2.**
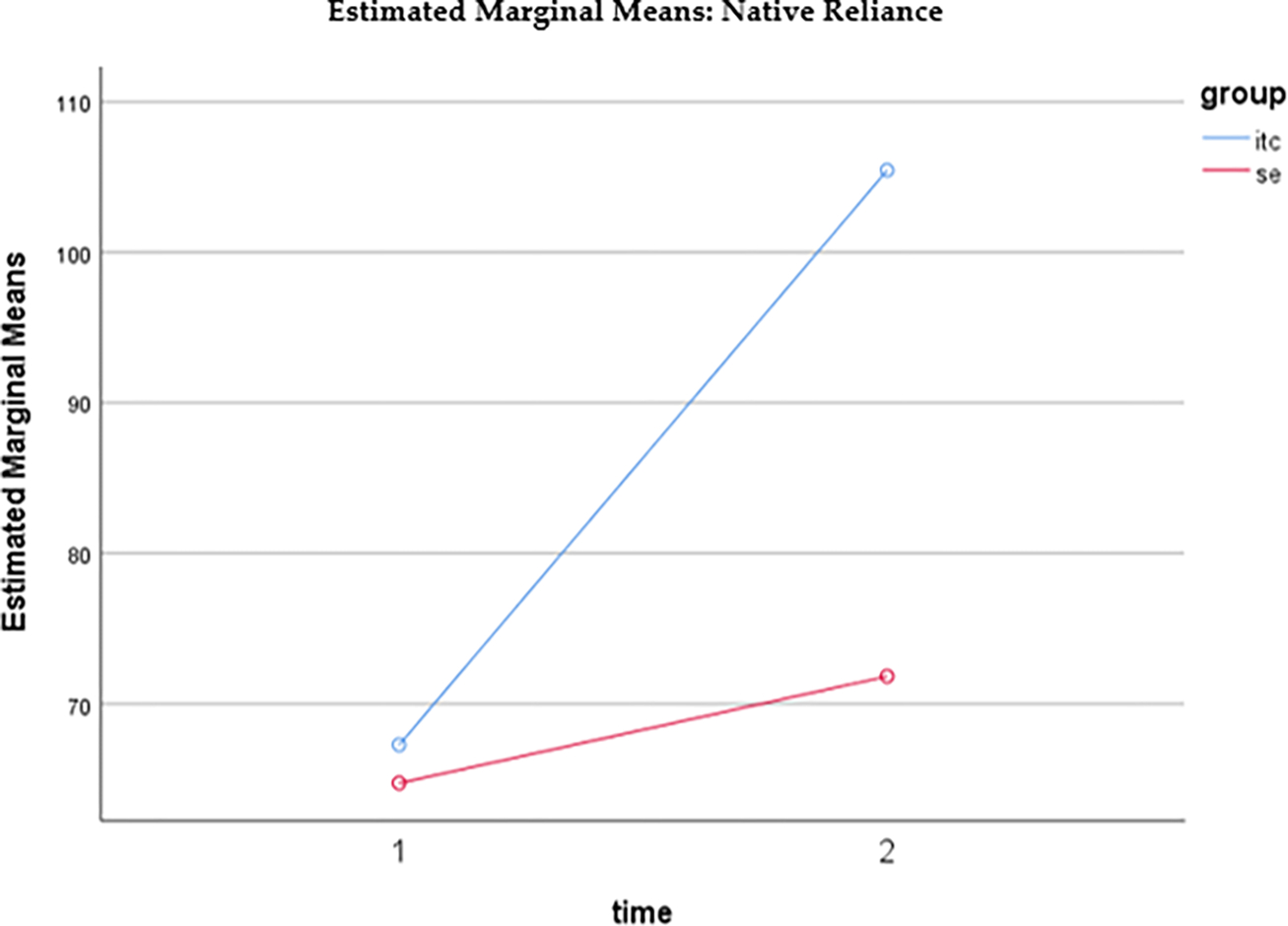
Estimated marginal means of Native Reliance

The mean score for cultural identity measured by the Native-Reliance questionnaire for the SE participants at pre-intervention (baseline) was 64.72 (SD = 14.054). The mean score for the SE participants at post-intervention was 71.82 (SD = 7.984). The mean scores for the UTC participants at pre-intervention (baseline) 67.26 (SD = 10.069) and at post-intervention was 105.44 (SD = 7.984). Both the within- and between-subject effects were significant. There was also a significant interaction between time and group. As displayed in Figure 2, at baseline (time 1), the two groups’ Native-Reliance scores were not significantly different (*t* = 1.039, *p* = 0.301). At post intervention (time 2), the UTC had a much higher Native-Reliance score than SE (*t* = 21.101, *p* < 0.001). For both groups, the Native-Reliance scores increased over time from pre to post. However, the UTC group (*t* = 19.888, *p* < 0.001) had a larger increase than the SE group (*t* = 4.248, *p* < 0.001).

### 5.2. Drug Use Interest

**Figure 3.**
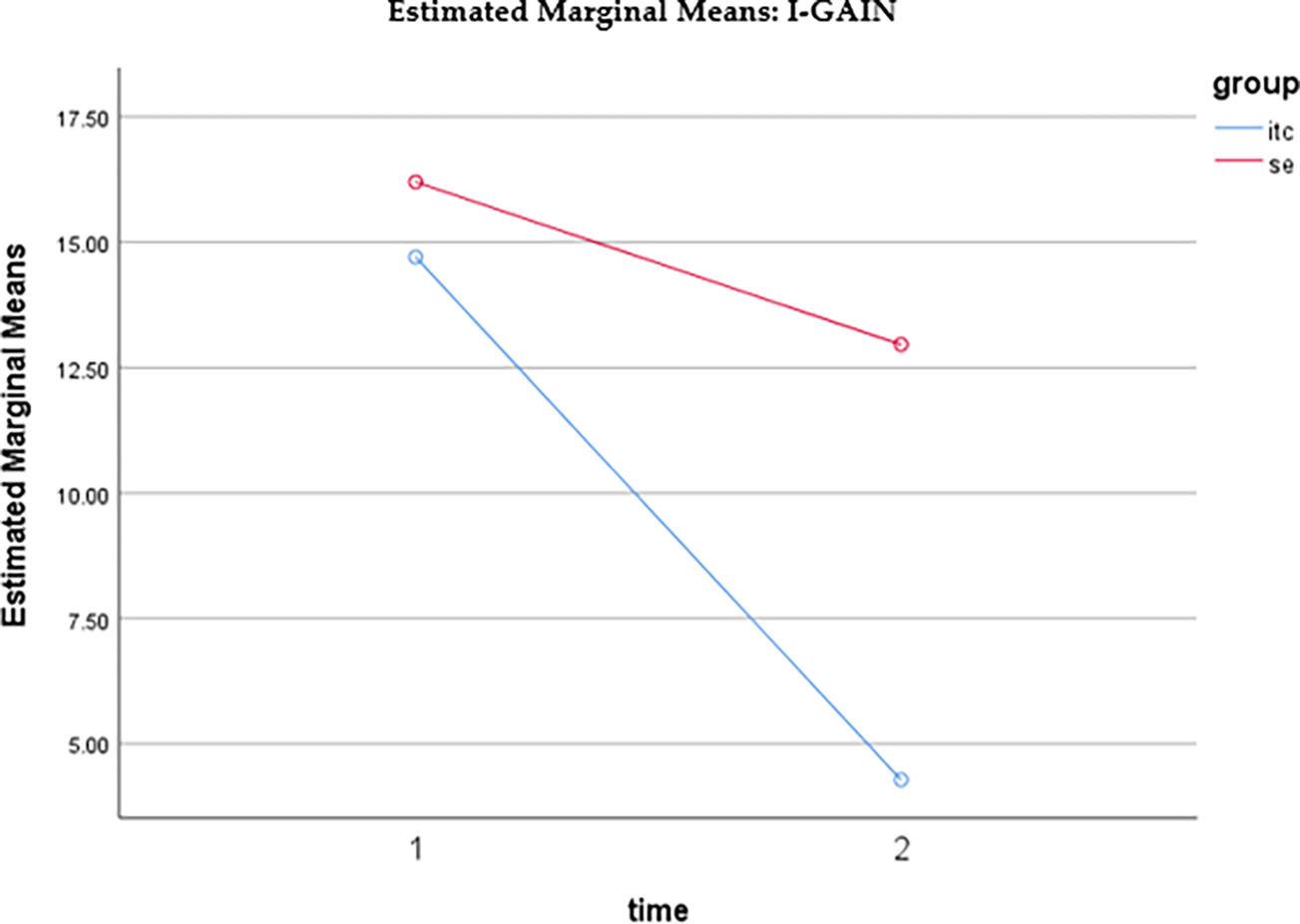
Estimated marginal means of I-GAIN

The mean score related to drug use interest measured by the Indigenous—Global Assessment of Individual Needs (I-GAIN) for the SE participants at pre-intervention (baseline) was 16.2000 (SD = 10.63111) and the mean score for the UTC participants at pre-intervention (baseline) was 14.7000 (SD = 7.59498). The mean score for the SE participants at post-intervention was 12.9600 (SD = 7.92815) and the mean score for the UTC participants at post-intervention was 4.2800 (SD = 4.69494). Both the within- and between-subject effects were significant. There was also a significant interaction between time and group. As displayed in Figure 3, at baseline (time 1), the two groups’ I-GAIN scores were not significantly different (*t* = 0.812, *p* = 0.419). At post-intervention (time 2), UTC had a much lower score than SE (*t* = −6.661, *p* < 0.001). For both groups, the I-GAIN scores decreased over time from pre to post. However, the UTC group (*t* = −16.180, *p* < 0.001) had a larger decrease than the SE group (*t* = −5.484, *p* < 0.001).

### 5.3. NAAMY

**Figure 4.**
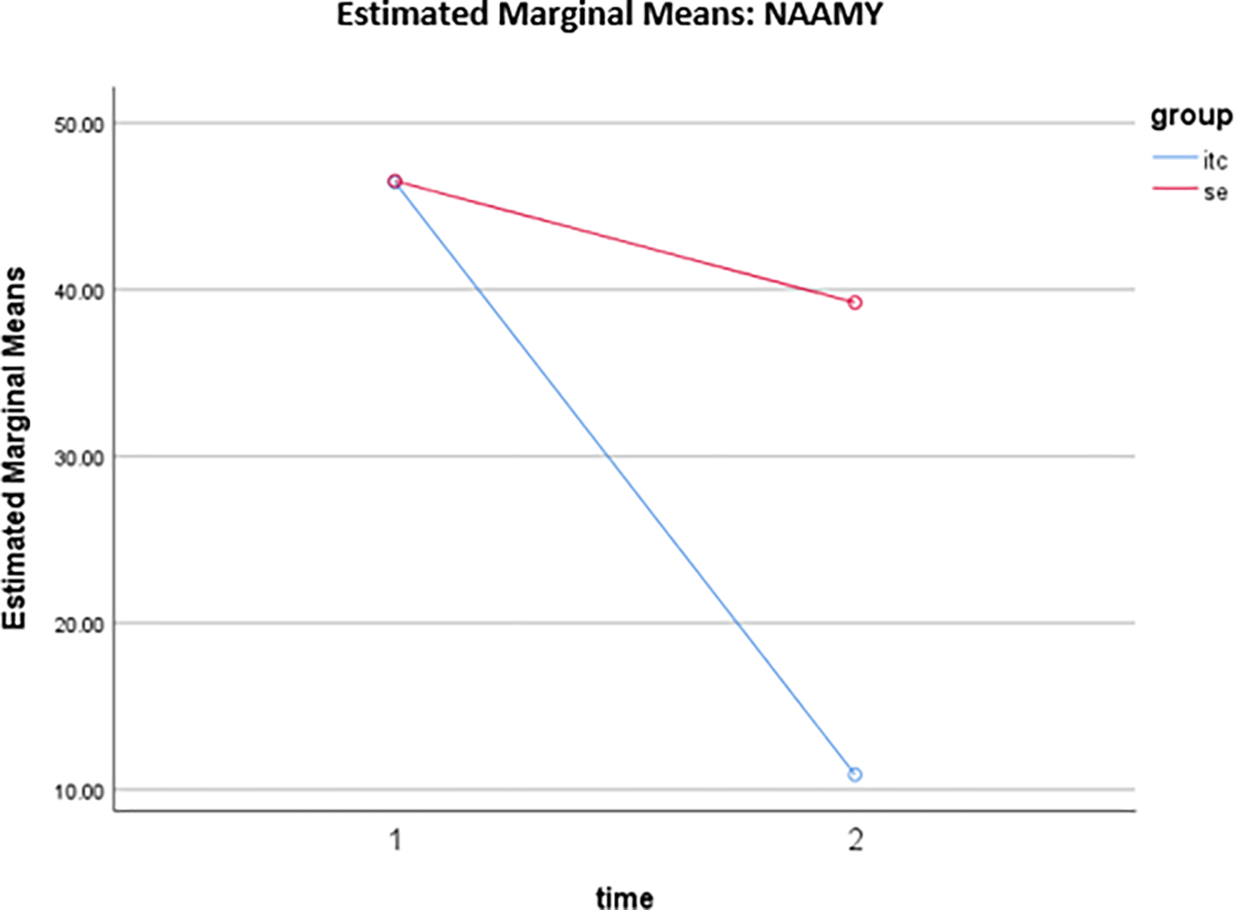
Estimated marginal means of NAAMY

The mean score regarding alcohol use interest assessed by the Native American Alcohol Measure for Youth (NAAMY) instrument at pre-intervention (baseline) was 46.5200 (SD = 21.64768) for the SE participants, and for the UTC participants at pre-intervention (baseline) was 46.4400 (SD = 14.08887). The mean score for the SE participants at post-intervention was 39.2200 (SD = 20.55385), and for the UTC participants at post-intervention was 10.9000 (SD = 8.56964). The NAAMY scores reveal that both the within- and between-subject effects were significant. There was also a significant interaction between time and group. As demonstrated in Figure 4, at baseline (time 1), the two groups’ NAAMY scores were not significantly different (*t* = −0.022, *p* = 0.983). Post intervention (time 2), UTC had a much lower NAAMY score than SE (*t* = −8.993, *p* < 0.001). For both groups, the NAAMY scores decreased over time from pre to post. However, the UTC group (*t* = −15.016, *p* < 0.001) had a larger decrease than the SE group (*t* = −8.894, *p* < 0.001).

## 6. Discussion

The findings of this study help to address some of the gaps concerning intervention studies for the prevention of alcohol and drug use among urban Native American youth populations. Research gaps include: (a) the lack of awareness and visibility regarding the need to study alcohol and drug use prevention among urban Native American youth; (b) findings that indicate how cultural protective factors, cultural identity, cultural safety, and humility play an important role in the prevention of alcohol and drug use among urban Native American youth; and (c) development and testing of cultural appropriate interventions for the prevention of alcohol and drug use among urban Native American youth populations. There is a definite need for conducting further studies that address culturally relevant interventions for the prevention of alcohol and drug use among this underrepresented and underserved population. Therefore, researchers must consider how to adapt scientific research approaches for the prevention of alcohol and drug use that are culturally relevant for urban Native American youth populations.

The research design in this study provided a collective, culturally relevant, yet holistic mechanism that tested the study aims and hypotheses. The integration and assessment of urban Native American cultural values and beliefs served to demonstrate the relevance of culture as a protective factor for the prevention of alcohol and drug use among southeastern urban Native American early adolescent youth.

The Native-Reliance theory guided the UTC intervention sessions. The integration and assessment of urban Native American cultural values and beliefs served to demonstrate the relevance of culture/cultural values and beliefs as protective factors against alcohol and drug use among this population. The incorporation of cultural factors into prevention efforts can enhance the acquisition of culturally relevant coping skills and, ultimately, lead to a reduction in substance use. Native Americans have long used the talking circle to celebrate the sacred inter-relationship that is shared with one another and with their world. The UTC intervention integrated concepts and values from the Native-Reliance model which describes the holistic worldview, values, beliefs, and behaviors among Native Americans.

Alcohol and drug use behaviors by urban Native American youth are rooted in historical, current, and cultural dispossession events. Urban Native American populations are experiencing a perpetual cycle of historical trauma related to loss and dispossession (Evans-Campbell 2008; Reinschmidt et al. 2016; Walters et al. 2002). Previous events related to assimilation, war, atrocities, relocations, and historical trauma often have resulted in urban Native American populations becoming invisible and vulnerable for health disparity issues such as alcohol and drug use. Within the general population of U.S. urban areas (e.g., California, Florida, New York, Texas), this invisibility among urban Native American people inhibits the recognition for culturally relevant alcohol and drug use prevention approaches (Chadwick and Stauss 1975; Robbins 1992; Urban Indian Health Commission and Robert Wood Johnson Foundation 2007). The problem of alcohol and drug use must also be addressed during the critical transition stage of early adolescence among urban Native American youth as the survival of urban Native American populations is dependent upon the health and well-being of its youth.

Quality assured, cultural appropriate, culturally safe, cost-effective, and collaborative community health prevention strategies need to be endorsed. More research focused on prevention programs for alcohol and drug use, depression, suicide, and other related conditions needs to be advocated for urban Native American populations. The results of studies that focus upon these types of prevention programs can provide evidence for culturally relevant, culturally safe, cost-effective, and collaborative community strategies among urban Native American populations.

Many health professions currently have transitioned from a focus of evidence-based practice to that of practice-based evidence within Native American settings (National Council of Urban Indian Health 2016). This includes the integration of cultural knowledge to approaches in delivering health care. The findings from this study provide evidence for the importance of providing cultural relevant knowledge and culturally safe approaches to alcohol and drug use prevention among urban Native American youth (Wilson and Neville 2009). Additionally, this study utilized a cultural relevant theory to guide the intervention approach for the prevention of alcohol and drug use among urban Native American youth. The intervention tested was congruent with Native American cultural values and beliefs and a two-condition quasi-experimental design was used to examine effectiveness of the UTC intervention among this population. Cultural considerations may enhance the level to which specific interventions address substance use and related problems among people from specific cultural groups. The importance of using an appropriate cultural model so that certain cultural constructs are presented and integrated was demonstrated in this study. The study approach also assured cultural safety by the operationalization of the cultural model throughout each phase of the study (Richardson et al. 2017). The research team was comprised of persons from the urban Native American community in Florida who could identify and address barriers to cultural adherence in an ongoing manner during the entire implementation of the study.

### Further Research

Recommendations for further intervention alcohol and drug use prevention studies include: (a) consideration for a two-condition longitudinal-experimental design; (b) inclusion of randomization of the study’s individual participants; (c) additional post-intervention assessment data collection points to assess longitudinal impact of the intervention; and (d) implementing and evaluating a training component of the UTC intervention where urban Native American community members, leaders, and others can be trained to sustain the intervention as an urban Native American youth community program.
